# Functional outcome following intracapsular resection of head and neck peripheral nerve sheath tumors: a retrospective cohort

**DOI:** 10.1186/s40463-023-00646-5

**Published:** 2023-10-03

**Authors:** Liyona Kampel, Marga Serafimova, Shaun Edalati, Adi Brenner, Razan Masarwy, Anton Warshavsky, Gilad Horowitz, Yuval Shapira, Nidal Muhanna

**Affiliations:** 1 The Department of Otolaryngology Head & Neck Surgery and Maxillofacial Surgery, The Interdisciplinary Center for Head & Neck Surgical Oncology, Tel Aviv Sourasky Medical Center, 6 Weizman St, 6423906 Tel Aviv, Israel; 2 The Department of Neurosurgery, Tel Aviv Sourasky Medical Center, Tel Aviv, Israel, Affiliated to the Sackler Faculty of Medicine, Tel Aviv University, Tel Aviv, Israel; 3 The Radiology and Neuroradiology Unit, Tel Aviv Sourasky Medical Center, Tel Aviv, Israel, Affiliated to the Sackler Faculty of Medicine, Tel Aviv University, Tel Aviv, Israel

**Keywords:** Schwannoma, Peripheral nerve sheath tumor, Intracapsular resection, *En bloc* resection, Intraoperative nerve monitoring

## Abstract

**Background:**

Intracapsular resection of head and neck peripheral nerve sheath tumors (PNST) has emerged as a nerve-preserving technique compared to *en bloc* resection. The aim of this study was to evaluate and compare the functional outcome of both surgical techniques performed at a single tertiary referral center.

**Methods:**

This is a retrospective cohort of patients with head and neck PNST undergoing surgical resection from 2011 to 2021 at the Tel Aviv Sourasky Medical Center. Demographic data, the nerve of origin and surgical technique, including the use of intraoperative nerve monitoring were recorded and analyzed in association with postoperative functional outcomes.

**Results:**

Overall, 25 patients who had a cervical or parapharyngeal PNST resected were included. Nerve function was preserved in 11 of 18 patients (61%) who underwent intracapsular resection, while all those who underwent *en bloc* resections inevitably suffered from neurologic deficits (100%, N = 7). Sympathetic chain origin and an apparent neurologic deficit pre-operatively were associated with postoperative neural compromise.

**Conclusion:**

Improved functional outcome can be anticipated following intracapsular resection of extracranial head and neck PNST compared to complete resection, particularly in asymptomatic patients.

**Graphical abstract:**

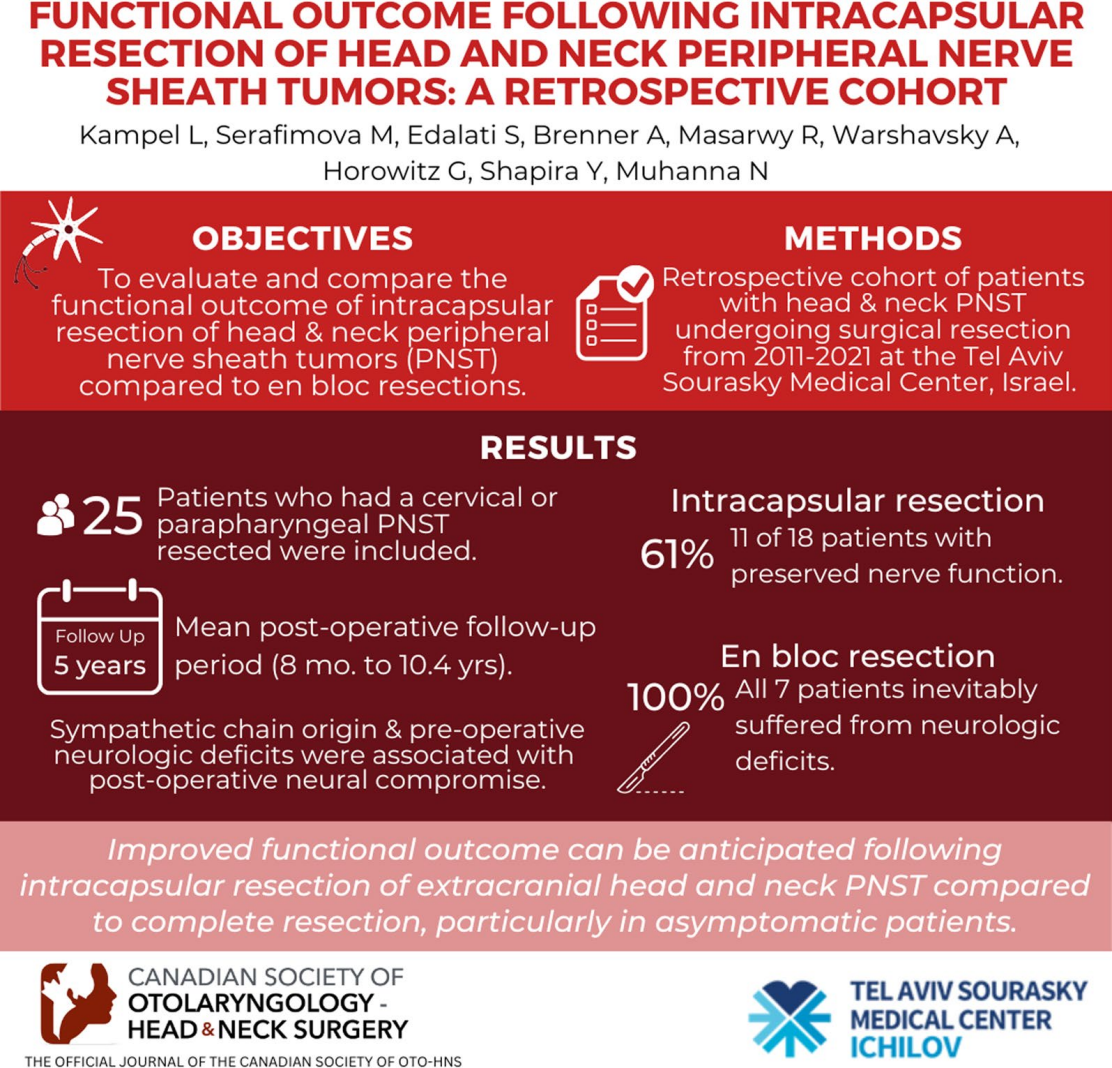

## Introduction

Peripheral nerve sheath tumors (PNST) comprise a group of neurogenic tumors that arise outside the central nervous system and are mostly benign. Extracranial schwannomas are the most prevalent PNST, and they arise from the neuro-lemmocytes (also known as Schwann cells) present in the head and neck in approximately 25–45% of cases [1]. Extracranial schwannomas are most often detected in the cervical or parapharyngeal region, where they typically affect either the vagus nerve or the sympathetic trunk [1].

Treatment of extracranial head and neck (ECHN) PNST varies according to the nerve of origin, the size of the tumor, and the patient’s surgical risk. Surgical resection remains the treatment of choice [2], but observation is widely accepted for asymptomatic cases due to the benign nature of these tumors [2, 3]. Most tumors will eventually be excised due to neural compromise, rapid growth or displacement and pressure they apply to adjacent structures.

The optimal surgical technique is a subject of controversy [1, 3]. *En bloc* resection results in complete excision of the tumor but carries the risk of neurologic deficit due to dissection of the involved nerve segment. Intracapsular resection attempts to preserve the nerve of origin without increasing the risk of tumor recurrence [4]. Intraoperative nerve monitoring has been applied for nerve preservation, but it does not guarantee the avoidance of neural deficit or the absence of residual tumor [3–6].

Several studies have evaluated the potential functional outcomes of intracapsular resection for PNST. Sandler et al. [1] conducted a comprehensive literature review on the diagnosis and management of cervical vagal schwannomas and discovered that using intraoperative nerve monitoring along with subtotal resection, improves nerve preservation compared to complete resection. Particularly in elderly patients or in patients with significant postoperative morbidity, subtotal resection was also preferred [1]. Another study by Jichi et al. [7], revealed that with tumor enucleation using an electromyographic system for intraoperative monitoring, nerve function was preserved in all 15 of their cases, and the frequency of nerve injury was reduced.

Although enucleation may have the advantage of nerve preservation and function, there may be an increase in exposure to local recurrence [8]. In a retrospective cohort study of 22 consecutive patients with schwannomas of the cervical vagus nerve, *en bloc* resection provided a durable, lifelong cure, whereas enucleation resulted in some tumor recurrence [8]. In this case, *en bloc* resection appears to be superior to enucleation as a curative method for treating schwannomas of the cervical vagus nerve [8].

Here, we explore the outcomes of surgical resection of ECHN PNST at the Tel Aviv Sourasky Medical Center (TASMC) during the past decade. Specifically the aims of the study are to assess the functional outcomes of intracapsular resection and identify patients who may benefit the use of nerve monitoring and microscope-guided micro-dissection technique.

## Materials and methods

### Study design and ethics

This is a retrospective cohort, performed at a single tertiary referral center. The study was approved by the TASMC institutional review board (IRB 0901–20-TLV), in accordance with the declaration of Helsinki.

### Patient selection and data collection

The medical records of all patients with histologically confirmed ECHN PNST resected at the TASMC between January 1, 2011, and December 31, 2021 were retrospectively reviewed. All patients included in the study underwent surgery. Patients being observed (who either refused surgery or were not considered for surgery) were not included. The medical records were retrieved from the hospital database, and the relevant clinical, radiological, and histopathological data were collected. Patients underwent preoperative ultrasound, computed tomography (CT), or magnetic resonance imaging (MRI) to determine the extent of the tumor and identify the nerve of origin. Fine-needle aspiration (FNA) cytology or an incisional biopsy was performed when imaging findings were inconclusive.

### Surgical approach

**Fig. 1:**
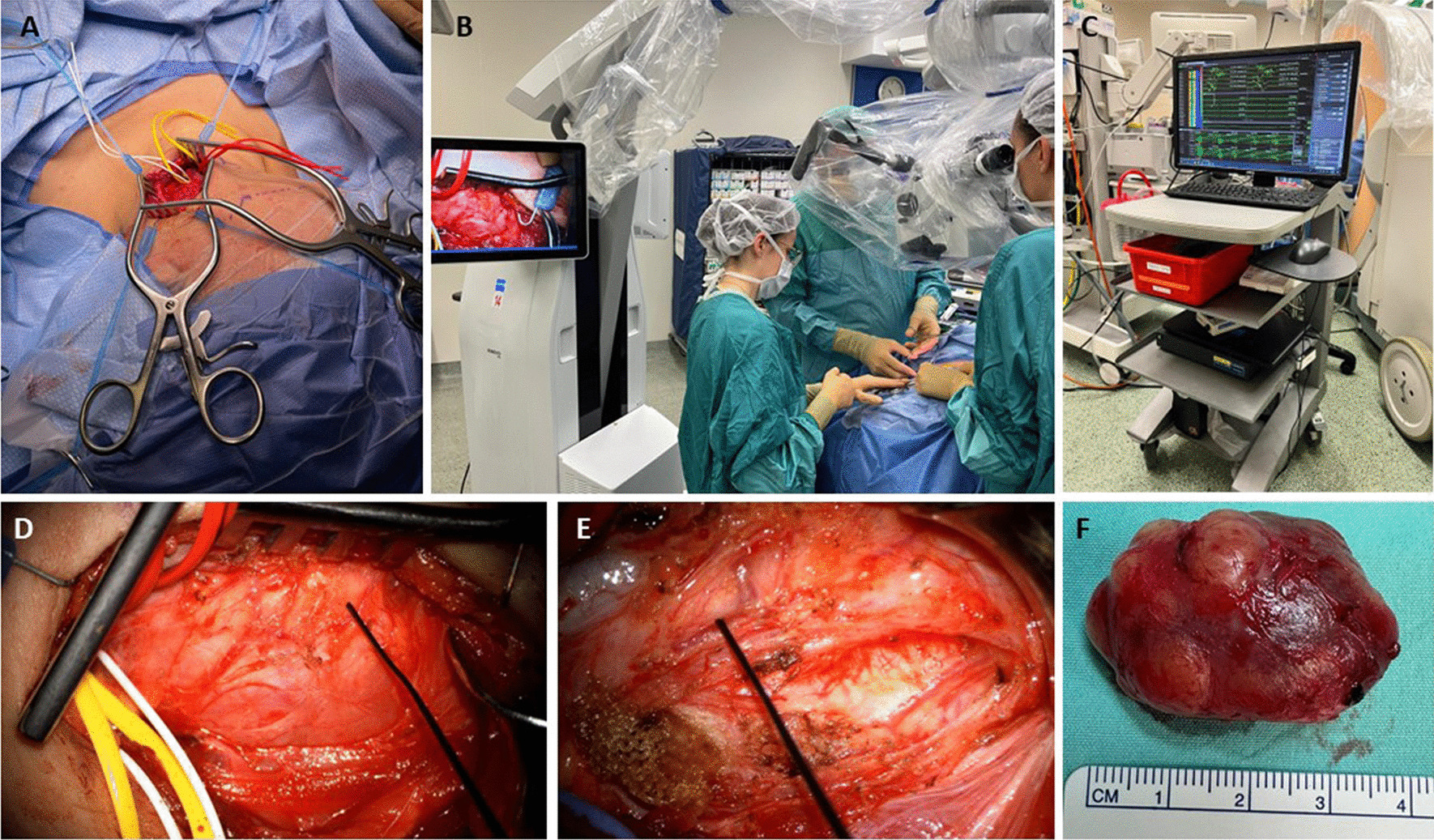
Vagal schwannoma intracapsular resection. **A** A vagal schwannoma displacing the jugular vein anteriorly was exposed via a transcervical approach. **B-E** The tumor capsule was carefully dissected under the guidance of a stimulus probe, an electrophysiology monitoring system, and a magnifying microscope. **F** Excised tumor

The surgical approach was determined by a multidisciplinary team consisting of head and neck surgeons, neurosurgeons specializing in peripheral nerve surgery, and radiologists. The surgical approach depended mostly upon the surgeon's expertise and preference. No clinical or radiological criteria were used to support the selection of any technique over the other, yet nerve-sparing intracapsular resection has emerged as the one most preferred. Intraoperative nerve monitoring with electrophysiology study, including endotracheal neural monitoring of the recurrent laryngeal nerve, were used during the resection of cervical and parapharyngeal tumors originating from motor nerves. After the tumor had been exposed, a stimulus probe was used to identify a safe location for dissecting the surface of the tumor without disrupting functional neural fibers. A surgical microscope and a stimulus probe were used to detect and outline tethered functional neural fibers while micro-dissecting the tumor, leaving its pseudo-capsule behind to avoid adjacent nerve fiber compromise (Fig. 1).

Patients were followed regularly at the otolaryngology or neurosurgery outpatient clinics after the surgery to document functional outcomes and recurrences. All patients were routinely invited for post-operative follow up at one month after surgery, and then once a year with an MRI study.

## Results

Twenty-five patients who underwent surgical resection of an ECHN PNST were included in the study. Table 1 lists their characteristics and the locations of the tumors. The cohort's age range was 18 to 81 years. Nineteen patients (76%) presented with a palpable painless mass. The mean diameter of all tumors was 40 mm (range 10-71 mm).

**Table 1:** Patient and disease characteristics

Characteristic	No. of patients (%)
Sex	
Male	15 (60)
Female	10 (40)
Age y, mean (range)	51 (18–81)
Location	
Parapharyngeal	13 (52)
Cervical	10 (40)
Facial	2 (8)
Mean size, mm (range)	40 (10–71)
Preoperative symptoms	
Painless mass	12 (48)
Odynophagia	2 (8)
Hoarseness	2 (8)
Sensory deficit/neuropathic pain	7 (28)
Incidental finding	2 (8)

A contrast CT or an MRI were obtained in all cases to confirm the diagnosis of suspected cervical or parapharyngeal lesions. While radiographic findings were usually sufficient to determine the origin of the tumor and its relation to major vessels, cytologic or histologic confirmation was important when considering the surgical approach. A preoperative FNA or a core biopsy was obtained in nine cases for which the diagnosis could not be established with certainty based upon the imaging studies, but they were diagnostic in only few of the cases. No complications related to biopsy procedure were recorded.

### Functional outcomes

The mean postoperative follow-up period was 5 years (range 8 months to 10.4 years). The functional outcomes of both surgical approaches were clinically evaluated. An intracapsular resection had been performed in 18 cases, and 7 patients underwent *en bloc* excision of the tumor (Table 2). Thirteen patients (52%) were left with a neurologic deficit, seven of whom (54%) had a neurologic deficit documented preoperatively. Four patients with a vagal schwannoma underwent intracapsular resection, and another 5 vagal schwannomas were completely excised by *en bloc* resection. Only one out of four intracapsular resections sustained vocal cord paralysis, while all five patients who underwent *en bloc* resection were left with hoarse voice due to vocal cord paralysis. Vocal cord mobility was evaluated post operatively by endoscopy in all cases.

**Table 2:** Surgical approach and outcome of extra-cranial head and neck PNST resection

Pt	Age (y)	Sex	Nerve of origin or location	Preoperative symptoms	Surgical approach	INM	Postoperative symptoms
1	28	F	Vagal	Cervical pain	Intracapsular	+	
2	35	F	Vagal	Painless mass	Intracapsular	+	
3	72	F	Vagal	Odynophagia	En bloc	–	Hoarseness, dysphagia
4	57	M	Vagal	Painless mass	Intracapsular	+	
5	38	M	Vagal	Painless mass	Intracapsular	+	Hoarseness
6	52	M	Vagal	Hoarseness	En bloc	–	Hoarseness
7	18	M	Vagal	Incidental	En bloc	–	Hoarseness
8	69	M	Vagal	Cervical pain	En bloc	–	Hoarseness, dysphagia
9	54	M	Vagal	Hoarseness	En bloc	–	Hoarseness
10	66	F	PPS	Odynophagia	En bloc	–	
11	47	F	PPS	Cervical pain	En bloc	–	
12	62	F	PPS	Painless mass	Intracapsular	–	
13	69	M	Brachial	Pain	Intracapsular	+	Radial numbness
14	65	M	Brachial	Numbness	Intracapsular	+	Radial numbness
15	63	F	Brachial	Pain	Intracapsular	+	C5-6 hypoesthesia
16	81	F	Brachial	Pain	Intracapsular	+	Neuropathic pain
17	37	M	Brachial	Pain	Intracapsular	+	
18	29	M	Brachial	Numbness	Intracapsular	+	
19	53	F	Cervical	Numbness	Intracapsular	+	
20	63	M	Cervical	Painless mass	Intracapsular	+	
21	33	F	Cervical	Painless mass	Intracapsular	+	
22	42	M	Sympathetic	Painless mass	Intracapsular	–	Horner syndrome
23	36	M	Sympathetic	Incidental	Intracapsular	–	Horner syndrome
24	50	M	Facial	Painless mass	Intracapsular	+	Facial palsy
25	30	M	Facial	Painless mass	Intracapsular	+	

PNST, peripheral nerve sheath tumor, INM, intraoperative nerve monitoring, PPS, parapharyngeal space

Two patients with schwannomas arising from the sympathetic chain developed ptosis and miosis (Horner syndrome). Three patients had a parapharyngeal space (PPS) lesion, in which the nerve of origin could not be determined (neither pre- nor intra-operatively). Interestingly, none exhibited neuronal dysfunction post-operatively, regardless of surgical approach, suggesting the nerve of origin was of negligible functional significance. Four out of six patients with brachial plexus neurofibromas sustained postoperative neurologic deficits despite the employment of intraoperative neural monitoring. Among patients whose nerve function was preserved following intracapsular resection, no functional deterioration was documented in follow up records.

### Residual disease

Only one patient had residual disease. She underwent an intracapsular resection of a vagal schwannoma under microscopic guidance and an intraoperative nerve monitoring system. The tumor was excised in a piecemeal fashion in order to avoid nerve dysfunction, which was successfully achieved. An MRI study obtained shortly after the surgery revealed a residual mass and the patient has been expectantly observed, with no neurologic deficit documented.

Fifteen patients (60%) adhered to the recommended surveillance paradigm and visited our outpatient clinic more than 1 year after tumor resection. No recurrences were documented, however the inadequate adherence to surveillance obviously limits recurrence rate evaluation.

## Discussion

ECHN PNST are rare benign tumors that commonly arise at the head and neck region. Although most patients with ECHN PNST are asymptomatic, rapid growth or neurologic deficits require definitive treatment in order to avoid further compression and subsequent ischemia of adjacent nerve fibers. Preoperative diagnostic imaging studies are essential for planning the optimal surgical approach and for determining the extent of surgical resection. There are two main approaches: complete tumor excision (*en bloc* resection) and intracapsular resection [3], and the risks and benefits of each need to be weighed according to the individual case [6]. In general, complete resection may ensure lower recurrence rates but it usually results in neural compromise, while intracapsular resection is a nerve-sparing technique but it bears the risk of residual or recurrent disease.

We assessed the functional outcomes of patients who underwent excision of ECHN PNST. Most of the patients in our cohort who underwent surgical resection of a cervical or parapharyngeal PNST (Table 2) were operated by means of a nerve-preserving technique. Intracapsular resection was guided by neural monitoring and an operating microscope for microdissection and preservation of functional nerve fibers. Outcomes were dependent upon several variables, including the location and size of the lesion and the ability to apply nerve stimulation during intraoperative monitoring.

Preservation of nerve function is challenging when the sympathetic chain is involved [7]. Both of the patients with sympathetic chain schwannomas in our cohort displayed ptosis and miosis after surgery. When the brachial plexus is the nerve of origin, preoperative neurologic deficit or neuropathic pain portends poorer functional outcomes, even with the use of intraoperative neural monitoring, however due to its functional significance, the intracapsular approach shall be the treatment of choice. We observed that excisions of cervical PNST originating from other nerves, less commonly resulted in postoperative neurologic deficits when applying the intracapsular resection technique. In our experience, the use of an intraoperative microscope and electrophysiologic monitoring resulted in nerve function preservation in nine out of 15 cases (60%). The nerve-preserving approach resulted in good functional outcomes without significant neural compromise compared to *en bloc* resections that unavoidably compromise nerve function by complete nerve transection. Notebly, even outside the cervical and brachial plexuses, nerve function preservation with intracapsular resection was successful in 5 out of 9 cases corresponding to 55%.

Patients with a solitary ECHN PNST should be considered as being at low risk of malignancy, although reports on recurrence rates are limited [7, 9]. No recurrences were documented among the patients in our cohort, while residual tumor was observed in one patient who underwent intracapsular resection.

We acknowledge the limitations of our study. This is a limited-number retrospective cohort of patients, underpowered to draw any statistically significant conclusions. Only minority of patients adhered to surveillance paradigm following the first year after surgery, noted by the median 1 year of follow up. This greatly limits recurrence rate determination. Still, this is one of the largest cohorts reporting the results of intracapsular microdissection of PNST in the head and neck region. Our data provide useful information for counseling patients considering definitive surgical resection for ECHN PNST about the potential function sparing. Longer follow-up studies are warranted, with timely imaging studies looking at the recurrence rates after surgical resection.

In conclusion, considering the benign nature of ECHN PNST, expectant observation may be suitable for asymptomatic patients. However, when surgery is required, intracapsular resection and intraoperative nerve monitoring may reduce complications and preserve functionality, especially when neuronal function has not been compromised by the tumor.

## Data Availability

The datasets used and/or analyzed during the current study are available from the corresponding author upon reasonable request.

## Abbreviations

PNST: Peripheral nerve sheath tumors
ECHN: Extracranial head and neck
TASMC: Tel Aviv Sourasky Medical Center
IRB: Institutional Review Board
CT: Computed tomography
MRI: Magnetic resonance imaging
FNA: Fine-needle aspiration

## References

[1] Sandler ML, Sims JR, Sinclair C (2019). Vagal schwannomas of the head and neck: a comprehensive review and a novel approach to preserving vocal cord innervation and function. Head Neck.

[2] Liu HL, Yu SY, Li GKH, Wei WI (2011). Extracranial head and neck Schwannomas: a study of the nerve of origin. Eur Arch Otorhinolaryngol.

[3] Yasumatsu R, Nakashima T, Miyazaki R, Segawa Y, Komune S (2013). Diagnosis and management of extracranial head and neck schwannomas: a review of 27 cases. Int J Otolaryngol.

[4] Sinclair CF, Tellez MJ, Roldan MAS, Urken M, Ulkatan S (2019). Intraoperative mapping and monitoring of sensory vagal fibers during vagal schwannoma resection. Laryngoscope.

[5] Biswas D, Marnane CN, Mal R, Baldwin D (2007). Extracranial head and neck schwannomas–a 10-year review. Auris Nasus Larynx.

[6] Shrikrishna BH, Jyothi AC, Kulkarni NH, Mazhar MDS (2016). Extracranial head and neck schwannomas: our experience. Indian J Otolaryngol Head Neck Surg.

[7] Ijichi K, Kawakita D, Maseki S, Beppu S, Takano G, Murakami S (2016). Functional nerve preservation in extracranial head and neck schwannoma surgery. JAMA Otolaryngol Head Neck Surg.

[8] Illuminati G, Pizzardi G, Minni A, Masci F, Ciamberlano B (2016). Superiority of resection over enucleation for schwannomas of the cervical vagus nerve: a retrospective cohort study of 22 consecutive patients. Int J Surg.

[9] Kim SH, Kim NH, Kim KR, Lee JH, Choi HS (2010). Schwannoma in head and neck: preoperative imaging study and intracapsular enucleation for functional nerve preservation. Yonsei Med J.

